# Climate protection, health and other motives for active transport – results of a cross-sectional survey in Germany

**DOI:** 10.1186/s12889-024-18609-4

**Published:** 2024-06-05

**Authors:** Ramona Moosburger, Kristin Manz, Almut Richter, Gert B.M. Mensink, Julika Loss

**Affiliations:** Department of Epidemiology and Health Monitoring, Robert Koch Institute, Berlin, Germany

**Keywords:** Mitigation, Climate change, Walking, Bicycling, Physical activity, Motivation, Nationwide survey, Adults

## Abstract

**Background:**

Active transport– for example walking and bicycling to travel from place to place– may improve physical fitness and health and mitigate climate change if it replaces motorised transport. The aim of this study is to analyse the active transport behaviour of adults living in Germany, to investigate differences among population groups and to determine whether climate protection is a frequent motive for this behaviour.

**Methods:**

This study uses self-reported data of 4,971 adults who participated in a national health survey (German Health Update 2021), which was conducted as a telephone survey from July to December 2021. Associations between active transport behaviour and corresponding motives with sociodemographic and health-related variables were analysed using logistic regression models.

**Results:**

Of the adult population, 83% use active transport at least once a week. The frequency and duration of walking per week are significantly higher than those for bicycling (walking 214 min/week; bicycling 57 min/week). Those with a lower education level are less likely to practise active transport than those with a higher education level. Furthermore, women are less likely to use a bicycle for transport than men. Among those practising active transport, the most frequently mentioned motive is “is good for health” (84%) followed by “to be physically active” (74%) and “is good for the climate/environment” (68%). Women and frequent bicyclists (at least 4 days/week) mention climate protection as a motive more often than men and those bicycling occasionally.

**Conclusions:**

The improvement of active transport, especially among people with lower education and women (for bicycling), may benefit from better insights into motives and barriers. Climate protection is an important motivator for practising active transport within the adult population living in Germany and should therefore have greater emphasis in behavioural change programmes.

## Background

Physical inactivity is a major public health issue because it is associated with many chronic diseases, such as coronary heart disease, several cancer types [1] and type 2 diabetes [2], and it contributes to many premature deaths worldwide. Regular physical activity can reduce the risk of those chronic diseases [3, 4]. Furthermore, any increase in physical activity is associated with a lower risk of premature mortality [5].

Active transport, also known as active travel, refers to modes of travel that involve a level of physical activity, also defined as “travel in which the sustained physical exertion of the traveller directly contributes to their motion” [6]. Active transport, for example walking or bicycling to travel from place to place, can contribute to an increase in physical activity on a daily basis. Moreover, active transport increases psychological well-being [7] and reduces stress [8]. Generally, it has been shown that the benefits of increased physical activity outweigh the risks of active transport, like traffic accidents and exposure to air pollution [9]. The transport sector is also linked to greenhouse gas emissions and global warming. As climate change has a significant impact on health, it has become an important topic in the public health community. In 2009, the Intergovernmental Panel on Climate Change (IPCC) defined climate change as the biggest global health threat of the 21st century [10].

Besides its health benefits, active transport can mitigate climate change if it replaces motorised transport. In 2019, the transport sector globally emitted approximately 8.7 gigatons of CO_2_ equivalents, making it the fourth-largest source of greenhouse gases (15%) [11]. One effective measure to decrease CO_2_ emissions is to reduce private motorised transport and replace it with active transport [12]. For example, a modelling study conducted in seven European cities estimated that, with every additional bicycle trip, life cycle CO_2_ emissions decrease by 14% and, with every person shifting their travel mode from car to bicycle, life cycle CO_2_ emissions decrease by 3.2 kgCO_2_/day on average [13].

Therefore, promoting active transport among populations can improve their physical fitness as well as their health and, as a “co-benefit”, can simultaneously contribute to the mitigation of climate change. In addition, the mitigation of climate change might reduce health risks on the global level. Therefore, replacing motorised transport with active transport can have direct and indirect health benefits. Active transport is also linked to other benefits, besides health and climate protection; for example, it is cheaper than driving a car and, for certain (shorter) distances, it can be faster than other transport modes. Furthermore, promoting active transport behaviour could contribute to make cities more liveable by reducing air/noise pollution and congestion and increasing road safety [14, 15].

In Germany, almost 20% of the annual CO_2_ emissions come from the transport sector, and 95% of these are from cars and trucks [16]. As Germany intends to achieve climate neutrality in the transport sector by 2045, comprehensive measures are required. There is considerable potential to increase active transport in Germany since 257 million journeys and over 3.2 billion passenger kilometres occur every day (2017) [17]: the majority of journeys (57%) by car, 10% by public transport, and only 22% on foot and 11% by bicycle. In German cities, 40–50% of car rides are shorter than five kilometres [18], a distance for which a bicycle can be a faster vehicle than a car or public transport.

For the conception of promotion strategies, it is important to know how widespread active transport currently is and which determinants play a role in its distribution. Furthermore, it is necessary to identify the advantages of active transport that motivate people to practise it.

A systematic review of reviews investigated the facilitators of transport-related physical activity [19]. The authors found some evidence of moderate consistency regarding a positive association between active travel behaviour and beliefs about the physical activity consequences (attitude and perceived benefits), walkability and existence of facilities that support active travel. No consistent association was found for social environment and interpersonal factors (like social norms and perceived safety).

An Europe-wide survey performed in 2022 found that 87% of Europeans had walked for active transport on at least one day in the past week for at least 10 min [20]; 61% did so on four to seven days. The proportion of people walking decreased with their age and increased with the community size. This survey also gave details for participants from Germany, who walked more often at least once a week than the average participants in the European countries, namely 93% (73% on at least four days). A German study of 2014/15 showed that around 80% of adults either walk or bicycle for transport at least once a week [21].

The report “Mobility in Germany” from 2017 showed that more men than women bicycle at least once a week [17]; additionally, they found that men cover longer distances on average by bicycle than women. However, it was found that women are less mobile in general; hence, they also use a car significantly less often. The finding that women are less likely to bicycle for transport than men has also been reported in several other studies [22–24].

The literature on a possible social gradient of active transport is inconsistent [25]. Some studies have found that higher educated people, or people living in high socioeconomic neighbourhoods, are more likely to walk and bicycle to travel from place to place than lower educated people, or people living in low socioeconomic neighbourhoods [26–31], while others have found the exact opposite [21, 31, 32].

Overall, the literature on climate protection as a motivator for active transport among adults is scarce. Nevertheless, it is quite feasible that environmental awareness can have an influence on individual transport choices. A study conducted among students, for example, finds that informing students about the environmental issues of different transport modes increases their likelihood of using sustainable mobility and can lead to a decrease in private transport usage [33]. Two surveys carried out in the Chinese and Indian contexts also showed that a high individual level of environmental awareness increases the likelihood of walking or bicycling [34, 35]. Another study performed among German students found that the personal contribution to reducing air pollution is a motivator to engage in active transport behaviour for more than half of the students [36]. The report “Monitoring of bicycling behaviour in Germany” (2021) [37] investigated individual reasons for advocating different transport modes. Environmental protection was often chosen as a reason for bicycling (47%) and for walking (56%). Other frequently chosen advantages of active transport were health (44% for bicycling, 67% for walking), costs (36% for bicycling, 39% for walking), flexibility and fun [37]. Climate protection in particular has been discussed increasingly in the media in recent years, but the role that climate protection plays, alongside other reasons as a motive for engaging in active transport, has not been well investigated.

Therefore, the aims of this study were to describe the active transport (walking and bicycling) behaviour of the adult population in Germany using population-wide representative data and to investigate the differences in the amount of active transport in relation to individual (gender, age, educational and health status) and contextual (community size) factors. In addition, the motives of individuals who were already travelling distances actively were investigated. The obtained insights may answer the following research questions: What differences are there in the distribution of active transport between defined population groups? What are the main reasons for this behaviour? Are there differences in the motives among the population groups? This information may provide implications for targeted policies to improve the use of active transport.

## Methods

### Study design and participants

The “German Health Update” (GEDA) study is being conducted regularly as part of the nationwide health monitoring at the Robert Koch Institute and aims to describe the health situation, health behaviour and its influencing factors and the use of prevention and care [38]. For GEDA 2021, a sample of randomly generated landline and mobile telephone numbers (dual-frame method) was drawn [39]. For households consisting of several people, the so-called Swedish Key was used to select participants randomly [40]. Participants were informed about the data protection policy, and informed consent was obtained verbally. The Ethics Committee of the Charité– Universitätsmedizin Berlin assessed the ethics of the study and approved its implementation with verbal informed consent (application number EA2/201/21). In this analysis, cross-sectional data that were obtained from 14 July to 30 December 2021 using computer-assisted telephone interviews are used. The present analysis covers 4,971 persons (51.0% female), who are representative of the population aged 18 years and above living in private households in Germany.

### Instruments and indicators

#### Frequency and duration of active transport

Active transport behaviour was assessed using the European Health Interview Survey– Physical Activity Questionnaire (EHIS-PAQ) [41]. The participants were asked on how many days per week they walk or bicycle for at least 10 min without interruption to travel from place to place.

People who stated that they use active transport at least once a week were asked about the usual duration on a typical day, using five answer categories (10–29; 30–59; 60–119; 120–179 and ≥ 180 min). A mean value for each category was assigned: 20, 45, 90, 150 and 210 min, respectively. To calculate the weekly duration of active transport, the mean duration of active transport on a typical day was multiplied by the number of days on which active transport took place (separately for walking and bicycling). People who stated that they used active transport less than once a week were coded with a weekly duration of 0 min.

### Motives for active transport

Those who practise active transport at least once a week were asked to indicate their motives for doing so (*n* = 4,232). There were seven motives to choose from (multiple answers possible): to be physically active, it is fast, it is cheap, it is good for health, it is good for the climate/environment, to avoid public transport and other reasons.

To evaluate the motives according to the frequency of walking or bicycling, the participants were divided into two groups: frequent walkers/bicyclists (4–7 days/week) and occasional walkers/bicyclists (1–3 days/week).

### Covariates

The analyses are presented stratified by gender, age group in years (18–29; 30–44; 45–64 and ≥ 65), education level, community size (< 5,000; 5,000 - <20,000; 20,000 - <100,000 and ≥ 100,000 inhabitants) and subjective health status of the participants. The participants were divided into three educational groups according to the International Standard Classification of Education (ISCED): low, medium and high [42]. Participants were asked to indicate their overall health status from very good to very poor (five categories). For the analyses, the categories were dichotomously summarised as “(very) good” and “moderate to (very) poor”.

The included variables contained some missing values (*n* = 15 for gender, *n* = 22 for education level, *n* = 300 for community size, *n* = 32 for walking, *n* = 16 for bicycling and *n* = 33 for motives). Since their number is low compared with the total number of participants, we applied no missing imputation. For the multivariable analyses, complete cases were used.

### Statistical analyses

The frequency of walking and bicycling as modes of active transport and the typical duration of active transport were analysed. The prevalence of people using active transport at least once a week was stratified by gender, age group, education level, community size and subjective health status. For the prevalence of the confirmed motives for active transport, stratified analyses by gender, age group and education level as well as occasional vs. frequent bicycling were performed.

The results are reported as prevalence in percentages with 95% confidence intervals (CI). To test the significance of group differences, chi-square tests and logistic regression analyses were performed. Through logistic regression analysis possible mediating effects of other determinants were investigated and adjusted for. For those analyses, odds ratios with a 95% CI and/or adjusted p-values are presented.

For all the analyses, a weighting factor was used to correct for different selection probabilities of mobile and landline phones. Furthermore, the weighting factor adjusted for deviations of the study participants from the official population structure in Germany considering age, gender, federal state, district type (as of 31 December 2020, Federal Statistical Office) and education level (micro census, 2018). The data were analysed with the survey procedures in the statistical software SAS (version 9.4). A difference between groups is considered to be statistically significant if the corresponding p-value is < 0.05.

## Results

Table 1 shows both weighted and unweighted characteristics of the study participants.

**Table 1:** Characteristics of the study participants (*n* = 4.971)

Study participants characteristics		N (unweighted)	% (unweighted)	% (weighted)
**Gender**	Female	2,583	51.8	51.0
	Male	2,373	47.9	48.2
**Age groups**	18–29 years	406	8.2	16.0
	30–44 years	795	16.0	22.7
	45–64 years	1,957	39.4	34.9
	65 + years	1,813	36.5	26.4
**Education level**	low	240	4.8	17.9
	medium	2,090	42.0	56.1
	high	2,619	52.7	25.3
**Community size**	< 5,000 inhabitants	1,098	23.5	27.4
	5,000-<20,000 inhabitants	928	19.8	21.0
	20,000-<100,000 inhabitants	1,052	22.5	23.5
	≥ 100,000 inhabitants	1,593	34.2	28.2
**Subjective health status**	(very) good	3,741	75.3	73.2
	moderate - (very) bad	1,230	24.7	26.8

15 missing for gender, 22 missing for education level, 300 missing for community size

### Frequency and duration of active transport

**Fig. 1:**
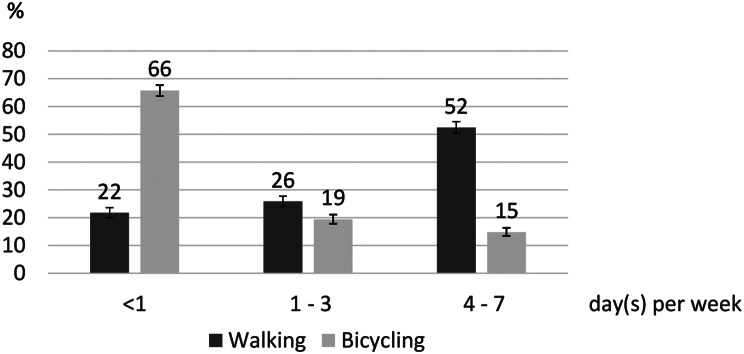
Frequency of walking and bicycling to travel from place to place. In days per week (in categories), *n* = 4,971. 32 missing for walking, 16 missing for bicycling

52% are frequent walkers and 15% are frequent bicyclists (on at least four days per week) (Fig. 1). Conversely, considerable proportions of adults never, or less often than once a week, walk (22%) and take the bicycle (66%) to travel from place to place. In total, 78% walk and 34% ride a bicycle at least once a week.

Adults walk on average for 214 min (CI 201–227) per week for active transport. The duration does not differ significantly between men (210 min, CI 190–229) and women (218 min, CI 200–236; *p* =.3066). Furthermore, adults bicycle for 57 min per week (CI 52–63) for transport. On average, men (71 min, CI 62–81) ride a bicycle for longer than women (44 min, CI 38–49; *p* <.0001).

Table 2 presents the prevalence of walking and bicycling at least once a week (yes/no) in different subgroups. The results of multivariable analyses adjusted for gender, age group, education level, subjective health status and community size are also shown in Table 2. In terms of walking for active transport, higher prevalences were found for women, young people, higher educated and subjective healthy adults as well as for people living in communities with over 100,000 inhabitants. Whereas women walk more often than men, they bicycle less often than men. Other groups with a significantly higher frequency of bicycling include people in the high education group, people living in places with 20,000 inhabitants and more and people with a better subjective health status. For bicycling as active transport, no significant differences between age groups were found.

**Table 2:** Prevalence of active transport (walking and bicycling) at least once a week, *n* = 4,971

Walking as active transport at least once a week (yes)									
					Binary analysis	Multivariable analysis			
		%	**confidence** **interval**		p-value*	Odds Ratio	confidence interval		adjusted p-value**
**Total**		78.3	76.4-80.0						
**Gender**	women	79.2	76.6-81.6			*reference*			*reference*
	men	77.0	74.2-79.5		0.2362	0.744	0.594-0.932		**0.0101**
**Age group**	18–29 years	86.5	81.3-90.5			*reference*			*reference*
	30–44 years	81.0	76.7-84.7			0.649	0.385-1.094		0.1045
	45–64 years	75.4	72.2-78.4			0.551	0.339-0.894		**0.0158**
	65 + years	74.6	71.3-77.6		**0.0002**	0.518	0.322-0.834		**0.0068**
**Education level**	low	74.2	67.6-79.9			*reference*			*reference*
	medium	77.6	75.2-79.9			1.270	0.851-1.895		0.2411
	high	82.2	80.1-84.1		**0.0205**	1.565	1.038-2.360		**0.0325**
**Community size**	< 5,000 inhabitants	72.8	68.7-76.5			*reference*			*reference*
	5,000-<20,000 inhabitants	78.2	73.7-82.1			1.271	0.923-1.749		0.1412
	20,000-<100,000 inhabitants	79.0	75.1-82.5			1.346	0.992-1.825		0.0564
	≥ 100,000 inhabitants	84.7	81.6-87.4		**< 0.0001**	1.887	1.387-2.566		**< 0.0001**
**Subjective health status**	(very) good	81.3	79.2-83.2			*reference*			*reference*
	moderate - (very) bad	70.0	65.9-73.7		**< 0.0001**	0.656	0.509-0.846		**0.0012**
**Bicycling as active transport at least once a week (yes)**									
					**Binary analysis**	**Multivariable analysis**			
		**%**	**confidence** **interval**		p-value*	**Odds Ratio**	**confidence interval**		**adjusted** p-value**
**Total**		34.2	32.3-36.2						
**Gender**	women	28.8	26.3-31.4			*reference*			*reference*
	men	39.7	36.7-42.7		**< 0.0001**	1.412	1.171-1.703		**0.0003**
**Age group**	18–29 years	36.7	30.7-43.2			*reference*			*reference*
	30–44 years	34.8	30.4-39.5			0.897	0.622-1.293		0.5589
	45–64 years	36.2	33.2-39.4			1.094	0.790-1.516		0.5878
	65 + years	29.6	26.6-32.8		0.0626	0.966	0.690-1.352		0.8385
**Education level**	low	25.4	19.8-31.9			*reference*			*reference*
	medium	32.8	30.2-35.6			1.396	0.941-2.073		0.0974
	high	43.2	40.6-45.8		**< 0.0001**	1.938	1.305-2.876		**0.0010**
**Community size**	< 5,000 inhabitants	28.5	24.8-32.5			*reference*			*reference*
	5,000-<20,000 inhabitants	35.8	31.4-40.6			1.249	0.948-1.645		0.1136
	20,000-<100,000 inhabitants	38.6	34.3-43.0			1.539	1.172-2.021		**0.0019**
	≥ 100,000 inhabitants	38.3	34.6-42.1		**0.0011**	1.420	1.103-1.830		**0.0066**
**Subjective health status**	(very) good	38.5	36.2-40.9			*reference*			*reference*
	moderate - (very) bad	22.4	19.0-26.1		**< 0.0001**	0.477	0.373-0.611		**< 0.0001**

15 missing for gender, 22 missing for education level, 300 missing for community size, 32 missing for walking, 16 missing for bicycling

Bold: significant difference

*chi²-test

**logistic regression analysis, adjusted for gender, age group, education level, subjective health status and community size; *n* = 4,700 (walking) and *n* = 4,711 (bicycling) complete cases were included in the multivariable analyses

### Motives for engaging in active transport

**Fig. 2:**
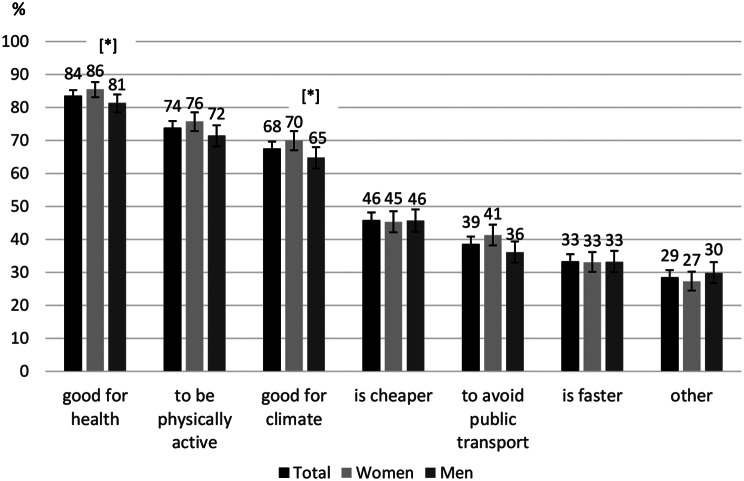
Motives for active transport in %. *n* = 4,199 (33 missing excluded). [*] significant difference between genders, adjusted for gender, age group and education level

Among the adult population, 83% (*n* = 4,232) report engaging in active transport (either walking or bicycling) at least once a week. This group was asked about the underlying motives for doing so. The majority chose several of the seven given motives (mean: 3.6 choices). “Because it is good for health” was the motive chosen most often (84%), followed by “to be physically active” (74%) and “because it is good for the climate/environment”, which was confirmed by 68% (Fig. 2).

The three most frequently mentioned motives were analysed in subgroups using logistic regression analyses, adjusted for gender, age group and education level (Table 3). The reason “it is good for health” was chosen more often by women and people aged 45 and older than by men and people aged 18–29. The motive “to be physically active” was also chosen more frequently by people in higher age groups: those aged 65 years and older mentioned this reason more often than those aged 18–29 years. Furthermore, this reason was chosen more often by people in the high education group than people in the low education group. There were no differences between the age and education groups concerning the motive “it is good for the climate/environment”, but women chose this reason significantly more often than men.

**Table 3:** The three most frequently chosen motives for engaging in active transport, stratified by gender, age group and education level, *n* = 4,199 (33 missing excluded)

		Good for health (yes)				To be physically active (yes)					Good for climate (yes)			
		%	CI	p-value*	adjusted p-value**	%	CI	p-value*		adjusted p-value**	%	CI	p-value*	adjusted p-value**
**Gender**	women	84.0	81.6–86.2		*reference*	74.6	71.8–77.3			*reference*	68.5	65.6–71.3		*reference*
	men	79.8	77.0-82.4	**0.0202**	**0.0480**	69.5	66.3–72.6		**0.0163**	0.0640	62.1	58.9–65.2	**0.0030**	**0.0213**
**Age group**	18–29	76.7	70.4–82.0		*reference*	65.9	59.0-72.2			*reference*	70.3	63.6–76.2		*reference*
	30–44	82.2	78.0-85.8		0.1506	71.7	66.6–76.3			0.3867	69.3	64.3–73.8		0.7023
	45–64	84.3	81.2–86.9		**0.0227**	73.4	69.8–76.7			0.1191	67.0	63.4–70.3		0.3603
	65+	88.5	85.9–90.6	**0.0004**	**< 0.0001**	81.8	78.7–84.5		**< 0.0001**	**< 0.0001**	64.9	61.1–68.5	0.4134	0.1609
**Education level**	low	81.4	74.1–86.9		*reference*	67.9	59.8–75.0			*reference*	67.4	59.4–74.5		*reference*
	medium	84.3	81.9–86.4		0.3826	73.2	70.3–75.9			0.1295	68.0	65.0-70.8		0.7159
	high	83.2	80.6–85.4	0.5656	0.5473	78.3	75.7–80.6		**0.0143**	**0.0026**	66.9	64.2–69.5	0.9224	0.8147

CI = confidence interval

Bold: significant difference

*chi²-test

**logistic regression analysis, adjusted for gender, age group and education level; *n* = 4,172 complete cases were included in the multivariable analyses

**Fig. 3:**
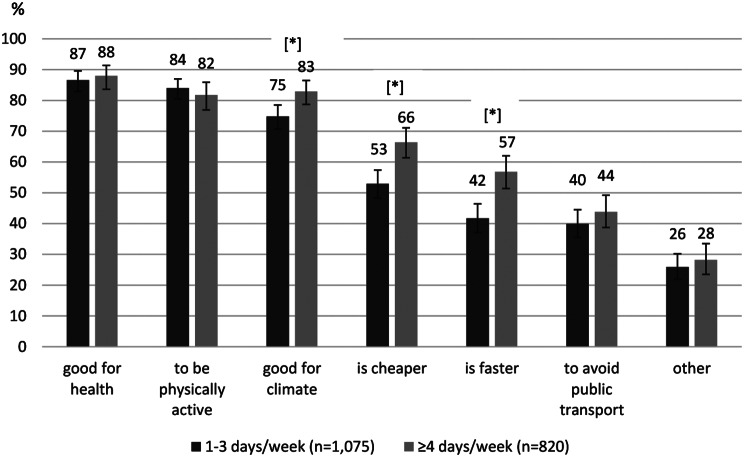
Motives for engaging in active transport by frequency of bicycling in %. *n* = 1,895. [*] significant difference, adjusted for gender, age group and education level

Frequent bicyclists (≥ 4 days/week) chose the motives “it is good for the climate/environment” (*p* =.0034), “it is cheaper” (*p* <.0001) and “it is faster” (*p* <.0001) more often as a reason for practising active transport than occasional bicyclists (1–3 days/week) (Fig. 3). In the group of frequent bicyclists, the climate motive was the second most chosen motive (83%) after health (88%) and before physical activity (82%). No significant differences in motives according to the frequency of walking (1–3 or ≥ 4 days/week; data not shown) were found.

## Discussion

### Summary of the main findings

More than 80% of adults living in Germany walk or bicycle at least once a week to cover distances. Walking is a more commonly used method of active transport, with about half of adults walking on at least four days per week. In comparison, a bicycle is used less often to cover distances, with only 15% of adults bicycling on at least four days a week. 66% use a bicycle less frequently than weekly for active transport. Whereas women walk more often than men, men bicycle more often than women to cover distances. Being higher educated, healthier and living in larger communities are associated with a higher frequency of both walking and bicycling for transport.

Active transport is generally motivated by a mixture of reasons, with health and physical activity benefits being the most frequent. With 68% practising active transport (also) because it is good for the climate and the environment, climate protection is a widespread motive, which was chosen more often by women and frequent bicyclists.

### Comparison with other studies

Compared with the Europe-wide survey mentioned in the background Sect. [20], the GEDA 2021 study observed a smaller proportion of adults walking as a mode of active transport. It must be mentioned that the participants in the cited European study were on average younger than the participants in the GEDA study. As found in the European study, the proportion of people walking decreased with age and increased with the community size.

In the GEDA 2021 study, similar proportions were observed for bicycling as were presented in the report “Mobility in Germany” (“Mobilität in Deutschland”) from 2017 [17]. Another German survey focusing on bicycle use (“Monitoring of bicycling behaviour in Germany” [Fahrrad-Monitor Deutschland], 2021) [37] found a higher percentage of adults riding a bicycle on several occasions per week, specifically 38%, than in the GEDA study, in which only 15% reported riding a bicycle on at least four days per week. Apart from the slight difference in categories, it should be noted that the data for “Monitoring of bicycling behaviour in Germany” were collected only in two consecutive summer months, when the prevalence of bicycling is known to be higher than during other seasons, and that the participants were generally younger (14–69 years) and hence probably more physically active than the participants in the GEDA study. The finding that women are less likely to bicycle as a form of active transport than men is in line with several other studies [22–25].

Previous studies have reported that the promotion and maintenance of health are an important motive for physical activity [43, 44] and specifically for bicycling [24, 35, 45, 46]. The GEDA 2021 study showed that this is also the case for active transport. Compared with the report “Monitoring of bicycling behaviour in Germany” [37], a higher percentage of adults living in Germany practising active transport for the reason of climate protection, health and costs was found. However, the comparability between these surveys is limited because, in the GEDA study, only participants who engaged in active transport were asked about their motives and no differentiation between walking and bicycling took place.

### Strengths and limitations

A strength of this study is that it was conducted as a nationwide representative survey of the adult population living in Germany. The prevalence of active transport and the role that climate protection plays alongside other reasons as a motive for active transport can therefore be generalised to the whole population. To the authors’ knowledge, this is one of the first studies to investigate the role of climate protection alongside other reasons as a motive for engaging in active transport on a national level. In addition, active transport behaviour was analysed in detail: frequency, duration and motives as well as associated factors were considered. Since the standardised questions of the European Health Interview Survey (EHIS) were used to assess the frequency and duration of active transport behaviour, the data are highly comparable with those of other European countries.

The study also has some limitations. First, other types of active transport, such as inline skating and travelling by pedal scooter, were not considered in this study. Other studies (e.g. EHIS) also focus on walking and bicycling as common types of active transport. In this respect the results are comparable. Future studies may also ask for further types of active transport to obtain a more comprehensive assessment and to analyse the specific contributions. Second, the specification within the question that “walking and bicycling should last at least ten minutes without interruption” might have led to an underestimation of the frequency and duration of active transport. A shorter duration may have neglectable impact on metabolism and health and would also complicate to summarize these activities for the participants. Third, the participants in the highest analysed age group were probably very heterogenic in their activity behaviour, since their age ranged from 65 to 97 years. Further differentiation of this age group was not possible due to the small number of people aged 80 years and above. To be able to investigate the active transport behaviour of older people in detail, specific study designs and recruitment approaches adapted to the needs of older people are necessary. Fourth, a selection bias cannot generally be ruled out as it can be assumed that health-conscious people are more likely to participate in health-related telephone surveys. The use of incentives may help reduce this bias in future surveys. Fifth, the patterns of outdoor physical activity are known to be influenced by weather conditions, and the GEDA 2021 study did not cover all seasons but had a time span of only six months, including summer, autumn and winter. This should be considered when comparing results. In general, covering a time span of a year is preferable for such surveys. Sixth, other motives for active transport that were not asked for in the survey also seem to play a role as almost 30% of participants (also) chose the category “other”. In future surveys other relevant motives may be determined through open questions. Last, it has to be considered that the GEDA 2021 study took place during the COVID-19 pandemic, which might have influenced active transport behaviour in different ways. There were no specific restrictions on active transport at the time of data collection. However, a study conducted in Germany in 2021 showed that 32% of the adult population had changed their active transport behaviour due to the pandemic (17% increased it and 15% reduced it) [47].

### Implications for policy and practice

The analysis shows that adults living in rural areas use active transport less often than adults living in urban areas, which, next to longer travelling distances, might indicate the lack of suitable infrastructure for active transport, especially in rural areas [48]. A systematic review of reviews, investigating the barriers to low-carbon transport mode adoption, found that infrastructure explains the largest amounts of differences in mode choice [25]. In order to promote active transport, it is important to provide a convenient and safe infrastructure for bicyclists and pedestrians, such as separate bicycle lanes. These route-level policies should be combined with society-level policies (e.g. lower speed limits for cars), city-level policies (e.g. car-free city centres) and individual-oriented policies (e.g. public campaigns) [49, 50, 51–53]. With 68% of adults living in Germany practising active transport to contribute to climate protection, this motive is certainly relevant and shows that climate protection can be used to motivate people to use bicycles or walk for transport. Politicians and decision makers should see this as support from the population to implement and expand sustainable infrastructure in favour of pedestrian and bicycle transport, as for example advocated in the sustainable urban mobility plans of some Italian regions/cities [54]. It could also be advisable to develop mass media campaigns that stress the environmental (and health) benefits of active transport [55]. Framing active transport as a multisolving behaviour that is an important means of obtaining both health and environmental benefits [56] may resonate well with many people as the analysis showed that the participants were often motivated by several reasons.

There is still a lack of evidence on the barriers that prevent people with lower education from engaging in walking or bicycling for active transport. When tackling health inequalities, it can be useful to invest in health-promoting and bicycling-friendly infrastructure and interventions in areas of higher deprivation in the city [57], as for example undertaken in the project “Cycling Coventry” [58, 59].

The importance of climate protection as a motive is apparently not dependent on age group and education level. This could be an indication that, among people using active transport, the climate crisis concerns all age and education groups in similar ways. However, in Germany, insights into peoples’ motives for engaging in active transport are scarce, and a more detailed picture of the population’s assessment of the climate crisis and individuals’ own willingness and engagement to mitigate climate change is needed.

## Conclusions

More than 80% of adults living in Germany practise active transport at least once a week. The following differences in the distribution of active transport between population groups were observed: people with higher education and people living in bigger cities are more likely to walk or bicycle for active transport than people with lower education and those living in smaller communities. The main reasons for practising active transport were health, physical activity and climate protection among the adult population living in Germany. These three reasons showed some differences among population groups and were more often mentioned by women, the reasons health and physical activity by older age groups and physical activity by those with a high education level. Promotion campaigns should therefore stress the multiple co-benefits of active transport. Future surveys should contribute to understanding travel behaviour choices, barriers to active transport (particularly in smaller and more deprived communities) and ways to overcome these barriers and motivate people to make climate friendly and health promotion choices.

## Data Availability

The data set cannot be made publicly available because the informed consent obtained from the study participants did not cover public deposition of data. However, the minimal data set underlying the findings is archived in the Research Data Centre at the Robert Koch Institute and can be accessed by researchers on reasonable request. On-site access to the data set is possible at the Secure Data Centre of the Robert Koch Institute’s Research Data Centre. Requests should be submitted by e-mail to fdz@rki.de.

## Abbreviations

IPCC: Intergovernmental Panel on Climate Change
GEDA: German Health Update
ISCED: International Standard Classification of Education
EHIS-PAQ: European Health Interview Survey - Physical Activity Questionnaire
CI: confidence interval
EHIS: European Health Interview Survey
